# Inhibition of *Trichinella spiralis* Membrane-Associated Progesterone Receptor (MAPR) Results in a Reduction in Worm Burden

**DOI:** 10.3390/vaccines11091437

**Published:** 2023-08-31

**Authors:** Muhammad Tahir Aleem, Zhaohai Wen, Zhengqing Yu, Cheng Chen, Mingmin Lu, Lixin Xu, Xiaokai Song, Xiangrui Li, Ruofeng Yan

**Affiliations:** 1 MOE Joint International Research Laboratory of Animal Health and Food Safety, College of Veterinary Medicine, Nanjing Agricultural University, Nanjing 210095, China; 2018207076@njau.edu.cn (M.T.A.); 2019207057@njau.edu.cn (Z.W.); 2020207062@stu.njau.edu.cn (C.C.); mingmin.lu@njau.edu.cn (M.L.); xulixin@njau.edu.cn (L.X.); songxiaokai@njau.edu.cn (X.S.); lixiangrui@njau.edu.cn (X.L.); 2 Center for Gene Regulation in Health and Disease, Department of Biological, Geological, and Environmental Sciences, College of Sciences and Health Professions, Cleveland State University, Cleveland, OH 44115, USA; 3 School of Agriculture, Ningxia University, Yinchuan 750021, China; yuzhengqing@nxu.edu.cn

**Keywords:** inhibitory effect, knockdown, rTs-MAPRC2-Ab, *Trichinella spiralis*, worm burden

## Abstract

*Trichinella spiralis* (*T. spiralis*), a nematode parasite, is the major cause of Trichinellosis, a zoonotic disease. A key role of MAPR in the reproductive system is to maintain pregnancy. Previous studies found that antihormone drug design and vaccine therapy of recombinant protein (rTs-MAPRC2) control *T. spiralis* infection. The current study investigates the inhibitory effects of different ratios of antibodies against Ts-MAPRC2 on the development of muscle larvae (ML) and newborn larvae (NBL). First, we performed indirect immunofluorescence assays and examined the effects of rTs-MAPRC2-Ab on ML and NBL in vitro as well as in vivo. Afterward, siRNA-Ts-MAPRC2 was transfected into *T. spiralis* muscle larvae. Following that, Ts-MAPRC2 protein was detected by Western Blotting, and mRNA levels were determined by qPCR. We also assessed whether siRNA-treated NBLs were infective by analyzing muscle larvae burden (MLs). Our results showed that rTs-MAPRC2-Ab greatly inhibited the activity of the Ts-MAPRC2 in ML and NBL of *T. spiralis* and rTs-MAPRC2-Ab reduced larval infectivity and survival in the host in a dose-dependent manner (1:50, 1:200, 1:800 dilutions). Furthermore, siRNA-Ts-MAPRC2 effectively silenced the Ts-MAPRC2 gene in muscle larvae (ML) in vitro, as well as in newborn larvae (NBL) of *T. spiralis* in vivo. In addition, siRNA-Ts-MAPRC2 (siRNA180, siRNA419, siRNA559) reduced host larval survival and infectivity significantly. This study, therefore, suggests that Ts-MAPRC2 might be a novel molecular target useful in the development of vaccines against *T. spiralis* infection.

## 1. Introduction

Trichinellosis is a zoonotic disease that is mainly caused by *Trichinella spiralis* (*T. spiralis*), a nematode parasite [1]. A significant source of infection with *T. spiralis* is pork byproducts, which are often eaten raw or undercooked [2,3,4]. China has a high morbidity rate linked to this disease due to its prevalence and the consumption of pork and pork products in the country [5,6,7]. The primary means of survival of *T. spiralis* nematodes is their ability to transmit directly from host to host. The immune system and normal cellular functions are adjusted to these infections at all stages [8,9]. Despite the use of antihelminthic agents against Trichinellosis, excessive usage leads to drug residues in meat, parasite resistance, and other environmental issues. To prevent the spread of Trichinellosis, it is critical to develop a vaccine that is effective for humans and pigs [7,10,11]. There have recently been discoveries of proteins that inhibit parasite viability, inhibit parasite invasion, and, thus, reduce susceptibility to parasite infection [12,13]. Additionally, their influence on *T. spiralis* larvae inoculation has been studied [10,11,14,15,16]. There have been some successes with the vaccines, but there is no equivalent vaccine to control *T. spiralis* infection [16]. A group of membrane-associated progesterone receptor (MAPR) proteins known as progesterone receptor membrane component 1 (PGRMC-1) and progesterone receptor membrane component 2 (PGRMC-2) belong to the same family (Membrane Associated Progesterone Receptor) [17,18,19,20,21]. Several studies reported the presence of PGRMC receptors, progesterone-induced proteins, progesterone receptor-associated proteins (p48 protein), and small androgen receptor-interacting proteins in *S. japonicum* [22,23]. Previously, we characterized and cloned the membrane-associated progesterone receptor component-2 (Ts-MAPRC2) gene from *T. spiralis* [24]. As part of the process, several experiments were conducted, including expression, purification, immunoblotting, binding ability against progesterone antibody, and immunofluorescence assay (IFA). Additionally, we evaluated the direct effects of progesterone (P4) and mifepristone (RU486) on Ts-MAPRC2 gene expression using in vitro cell culture tests, which revealed that expression levels varied at each stage of development (muscle larvae, female adult worms, male adult worms, and newborn larvae). Later, the in vivo phenotypic effects and relative mRNA effects of mifepristone were assessed in relation to the F-AL stage [24,25,26]. Small interfering RNAs (siRNAs) are double-stranded RNAs that carry 21–25 nucleotides and are found in *C. elegans* [27]. siRNAs are synthesized artificially and are used to study host–parasite interactions [28]. They negatively modulate gene expression. In the last decade, scientists have begun exploring siRNA as an efficient tool for identifying gene functions [29,30]. It can be used to silence the genes necessary for the development of parasites and the molting processes of *T. spiralis*, thus reducing parasite attacks on humans and other mammals [29,31]. In the current study, we investigate the inhibitory effect of rTs-MAPRC2-Ab ratios in the ML and NBL stages. Afterward, we performed indirect immunofluorescence assays (IIFA) at the ML and NBL stages. We also investigated the in vitro effects of rTs-MAPRC2-Ab on ML and NBL. Furthermore, we examined the infectivity of ML and NBL treated with rTs-MAPRC2-Ab in vivo. This study also focuses on preparing different groups treated with siRNA-Ts-MAPRC2 (siRNA-180, siRNA-419, siRNA-559, and siRNA-control, along with PBS control), and their transfection into *T. spiralis* muscle larvae. Afterward, we performed Western blotting of the Ts-MAPRC2 protein as well as the quantification of mRNA expression for Ts-MAPRC2. Additionally, we conducted an in vitro phenotyping study of the effects of siRNA on ML stages. Moreover, we evaluated the infectivity of NBLs treated with siRNA by examining the muscle larvae burden (MLs).

## 2. Materials and Methods

### 2.1. Parasite and Animals Preservation

Sprague–Dawley rats (body weight 220–250 g) and BALB/c mice (body weight 18–20 g) were purchased from Qinglongshan Animal Breeding Farm (SXK 2008–0004) in Nanjing, Jiangsu, China. At the Animal House of Nanjing Agricultural University, the animals were maintained under supervised conditions. In Nanyang, Henan Province, China, *T. spiralis* (ISS534) was isolated from a pig and maintained in BALB/c mice using serial passage every six to eight months. A standardized HCl-pepsin digestion protocol was used 40 days post-infection to recover muscle larvae (ML) of *T. spiralis* from the BALB/c mice [24]. As previously reported, adult worms (AL) were collected 6 days after ingestion, and newborn larvae (NBL) were collected from an adult female (F-AL) on RPMI-1640 culture media at 37 °C for 24 h [24]. Several development phases of parasites were collected and homogenized in liquid nitrogen.

### 2.2. Molecular Cloning of Ts-MAPRC2 and rTs-MAPRC2 Protein Production

Among its 234 amino acids, Ts-MAPRC2 has a full-length sequence of 705 bases. In the current study, a fragment with a size of 97–234 amino acids (225–705 bp) was expressed from the Ts-MAPRC2 with a conserved domain (104–173 amino acids). First, TRI-zol reagent was used to isolate total RNA from adult worms (Vazyme Biotech Co., Ltd., Nanjing, China). Next, the HiScript III First Strand cDNA Synthesis Kit was used to synthesize 1 μg of RNA into complementary DNA (cDNA) [32,33]. The Ts-MAPRC2 gene in *T. spiralis* was cloned and recombinant protein (rTs-MAPRC2) was produced using the method given in our previous research [24].

### 2.3. Obtaining Rat Polyclonal Antibodies against rTs-MAPRC2

We injected equal amounts of rTs-MAPRC2 protein (300 μg) and Freund’s complete adjuvant (Sigma-Aldrich, Darmstadt, Germany) subcutaneously into SD rats (n = 6) for preparation of antisera. In the second week, Freund’s Incomplete Adjuvant (Sigma-Aldrich) was used to inoculate the second dose (200 μg rTs-MAPRC2 protein). As before, we administered the two booster injections (200 μg rTs-MAPRC2 protein) at a one-week interval. For further experiments, serum samples were collected one week after the last dose and stored at −80 °C. Negative controls included serum collected from rats who were not treated (n = 3). In a previous study, we successfully characterized and cloned the Ts-MAPRC2 gene (480 bp), expressed it into pET-32a (expression vector), confirmed restriction enzyme digestion with EcoR I and Hind III, purified rTs-MAPRC2, prepared polyclonal antibodies, and performed immunoblot analysis [24,25].

### 2.4. Detection of the Native Protein Ts-MAPRC2 by Indirect Immunofluorescence Assay (IIFA)

As a first step, whole worms of ML (50 worms per group) and NBL (50 worms per group) were soaked in 5% BSA (Bovine serum albumin) to block nonspecific binding, then incubated with primary antibodies (rat sera against rTs-MAPRC2 (1:50, 1:200, and 1:800 dilutions)), pET32a rat serum (control group), and PBS (control group) at 37 °C for 2 h. Different incubation times (2 h, 4 h, 8 h, and 24 h) of ML were used to detect native Ts-MAPRC2 protein. Each group of worms (ML, NBL) was washed with PBS and incubated with FITC-anti-rat IgG conjugate (1:100; Santa Cruz, Dallas, TX, USA). After a further wash in PBS, the complete worms (ML, NBL) were observed under an Olympus fluorescent microscope (Tokyo, Japan) [11,34]. Relative fluorescent intensities were measured by Image J software and expressed in percentages.

### 2.5. In Vitro Phenotyping of rTs-MAPRC2-Ab Effects on ML and NBL

At the ML and NBL stages, the selected concentration rTs-MAPRC2-Ab ratios (1:50, 1:200, and 1:800 dilutions) were used in conjunction with controls pET32a rat serum (control group) and PBS (control group). A 6-well plate was used with 2000 worms/well of both stages (ML, NBL). Culture medium was composed of RPMI-1640, 10% heat-inactivated FBS (Fetal Bovine Serum), and 2% antibiotics (100 U/mL penicillin and 100 mg/mL streptomycin) (Gibco, Paisley, UK), and incubated at 37 °C with 5% CO_2_. ML stage parasites were tested at various time intervals (0 h, 4 h, 8 h, 24 h) for motility and ecdysis (molting process) following methods in the literature [33,34,35]. NBL motility was checked after 24 h. An inverted bright field microscope (Olympus, Shibuya, Japan) was used to examine the phenotypic appearance at both stages (ML and NBL) [24].

### 2.6. In Vivo Assessment of the Infectivity of ML Treated with rTs-MAPRC2-Ab

A total of fifty BALB/c mice (body weight 18–20 g) were acquired at Qinglongshan Animal Breeding Farm (SXK 2008–0004) in Nanjing, Jiangsu, China, and kept in animal housing at Nanjing Agricultural University. In order to determine the effectiveness of rTs-MAPRC2-Ab against *T. spiralis*, the mice were divided randomly into five groups. (n = 10 per group). To find out the infectivity of ML and efficacy of rTs-MAPRC2-Ab, three experimental groups (1:50, 1:200, and 1:800 dilutions), one pET32a rat serum (control group), and one PBS (control group) were infected orally with 500 MLs treated with rTs-MAPRC2-Ab (1:50, 1:200, and 1:800 dilutions), pET32a rat serum (control group) and PBS (control group), respectively. A total of five mice (n = 5) from each group were euthanized six days post-infection (6 dpi). Adult worms were collected from the mice intestines using the method discussed above, and were counted as follows: adult female (F-AL) (50 worms) length, adult male (M-AL) (50 worms) length, and total adult worm (AL) [24]. The number of NBL was also assessed and collected by culturing 50 adult female worms per group in RPMI-1640 with FBS (20%), penicillin (500 units/mL), and streptomycin (500 mg/mL) for 24 h at 37 °C with 5% CO_2_. MLs were collected from all five remaining mice in each group by digestion after euthanasia at 35 dpi [36].

### 2.7. Assessment of the Infectivity of NBL Treated with rTs-MAPRC2-Ab

Thirty mice were divided into 5 groups (n = 6 per group) and injected through tail vein injection with 20,000 NBLs treated with rTs-MAPRC2-Ab (1:50, 1:200, and 1:800 dilutions), pET32a rat serum (control group), and PBS (control group), respectively. The mice of every group were euthanized at 35 dpi, and the MLs were collected by artificial digestion as described above. Based on treatment with rTs-MAPRC2-Ab (1:50, 1:200, and 1:800 dilutions), and in comparison, to controls pET32a rat serum (control group) and PBS (control group), ML reductions were calculated [36].

### 2.8. Preparation of siRNA

To design siRNA sequences, full-length cDNA encoding Ts-MAPRC2 (705 bp) was used (Gene Bank accession XM_003375886.1) [37]. This study used Ts-MAPRC2-specific siRNA oligos created by Gene Pharma (Shanghai, China) (Stealth TM RNAi duplexes). As shown in Table 1, the sequences of three siRNAs and one control siRNAs were used in this study. 

### 2.9. Transfection of siRNA to T. spiralis Worms

The soaking method was used to knock down Ts-MAPRC2 gene expression [29,30]. The muscle larvae were cultured in RPMI-1640 (supplemented with penicillin 500 units/mL and streptomycin 500 mg/mL) with siRNA-180, siRNA-419, and siRNA-559 in 12-well culture plates (500 MLs/500 uL) at 37 °C with 5% CO_2_. After 36 h, 20% FBS was added and incubated for another 24 h. PBS and siRNA-Control were used as controls. After incubation with Lipofectamine 2000 Reagent (Invitrogen, US) for 20 min [38,39], control siRNA or siRNA-Ts-MAPRC2 were added to larvae in a final concentration of 2 μM [28].

### 2.10. Protein Expression of Ts-MAPR Determined by Western Blotting

In order to observe the effect of siRNA on the expression of Ts-MAPRC2, worms were harvested after 3 days of incubation. Proteinase inhibitor (Thermo Scientific, Waltham, MA, USA) and RIPA solution (Thermo Scientific, Waltham, MA, USA) were used for complete lysis. The total soluble protein of *T. spiralis* larvae (muscle larvae) parasites were collected from all treated groups (siRNA-180, siRNA-419, siRNA-559, siRNA-Control, and PBS control). The total soluble protein of *T. spiralis* was collected (high-speed centrifugation at 12,000 r/min for 30 min at 4 °C) and analyzed via the bicinchoninic acid method (BCA) previously described [33]. Then, the total soluble proteins of the *T. spiralis* (muscle larvae) parasites were separated by SDS-PAGE (12%) and transferred to polyvinylidene difluoride (PVDF) membranes (Millipore, Bedford, MA, USA). Next, nonspecific binding was blocked with 5% skim milk in TBST containing 0.1% Tween-20. After three washes with TBST, the membranes were incubated overnight at 4 °C with the primary antibody (rat sera against rTs-MAPRC2, 1:300 dilutions). Afterward, the membranes were washed three times and incubated for 1 h at 37 °C with HRP-conjugated goat-antirat IgG (diluted 1:5000 in TBST). Additionally, a mouse antibody against GAPDH (1:1000) (Proteintech, US) was used as a quantitative protein control to detect GAPDH expression. A secondary antibody was goat anti-mouse IgG (1:5000) HRP-conjugated. To detect bound antibodies, the Tanon™ High-sig ECL Western Blotting Substrate kit was used according to the manufacturer’s instructions [40]. Protein expression densities were measured by Image J software and expressed in percentages.

### 2.11. Expression of Ts-MAPRC2 mRNA by RNA Extraction and qPCR

In brief, the Trizol method was used to extract RNA from ML worms. A prime script RT reagent kit (Takara, CA, USA) was used for RNA extraction from siRNA-treated groups (siRNA-180, siRNA-419, siRNA-559, siRNA-Control, and PBS control) of ML worms. In order to reverse transcribe RNA isolated from each group at the ML stage, HiScript II Q RT SuperMix kit (Vazyme, Nanjing, China) was used. To analyze the Ts-MAPRC2 transcript level, the following primers were used: forward (5′-GCTGGGATTCTCGGAATAATGT-3′) and reverse (5′-CGGTGGACAATCTCCTGACAGTTG-3′). BI 7500 Fast Real-time PCR System (Applied Biosystems, Foster City, CA, USA) and Cham-QTM SYBR RT-qPCR master mix-Kit (Vazyme, Nanjing, China) were used for quantitative amplification. In order to ensure internal control, GenBank Accession No. AF452239 GADPH (Glyceraldehyde-3-phosphate dehydrogenase) of *Trichinella* was used. GADPH primers were designed as follows: forward (5′-GTCGTGGCTGTGAATGATC-3′) and reverse (5′-GCTGCCCCACTTAATTGCTT-3′), and data were computed using the comparative Ct (2−ΔΔCt) method [41]. 

### 2.12. In Vitro Phenotyping of siRNA Effects on ML

In 12-well culture plates (500 ML/500 μL) at 37 °C with 5% CO_2_, muscle larvae were cultured in RPMI-1640 (supplemented with penicillin 500 units/mL and streptomycin 500 mg/mL) with siRNA-180, siRNA-419, siRNA-559, siRNA-Control, and PBS control as described above [42]. ML stage parasites were tested for motility and ecdysis (molting process). An inverted bright field microscope (Olympus, Shibuya, Japan) was used to examine the phenotypic appearance [24].

### 2.13. Evaluation of siRNA-Treated NBL for Infectivity

Twenty-five mice were divided into five groups (n = 5 per group), and each group was infected using 20,000 NBLs treated with siRNA-180, siRNA-419, siRNA-559, siRNA-Control, and PBS control according to the procedure described above [38,39]. At 35 dpi, the mice of each group were euthanized, and the MLs were collected by artificial digestion as described above. The reductions in MLs were calculated based on treatment with siRNAs (siRNA-180, siRNA-419, siRNA-559) compared to siRNA-Control and the PBS control [36].

### 2.14. Statistical Analysis

The data were analyzed using analytical statistics software (Statistix 8.1, 2003). A one-way analysis of variance (ANOVA) was performed, followed by Tukey’s analysis and calculation of LSD (least significant difference). Standardized RT-qPCR data were analyzed in Microsoft Excel 2010 (Redmond, Washington, DC, USA) using 2−ΔΔCt. Triplicates of all tests were performed. Figures were created using Origin software (Origin Pro 2021, Origin Lab Corporation, Northampton, MA, USA). Data were expressed as mean ± SD (n = 5). *p* ≤ 0.05 was considered significant.

## 3. Results

### 3.1. Indirect Immunofluorescence Assay (IIFA) in Muscle Larvae (ML) and Newborn Larvae (NBL)

**Figure 1:**
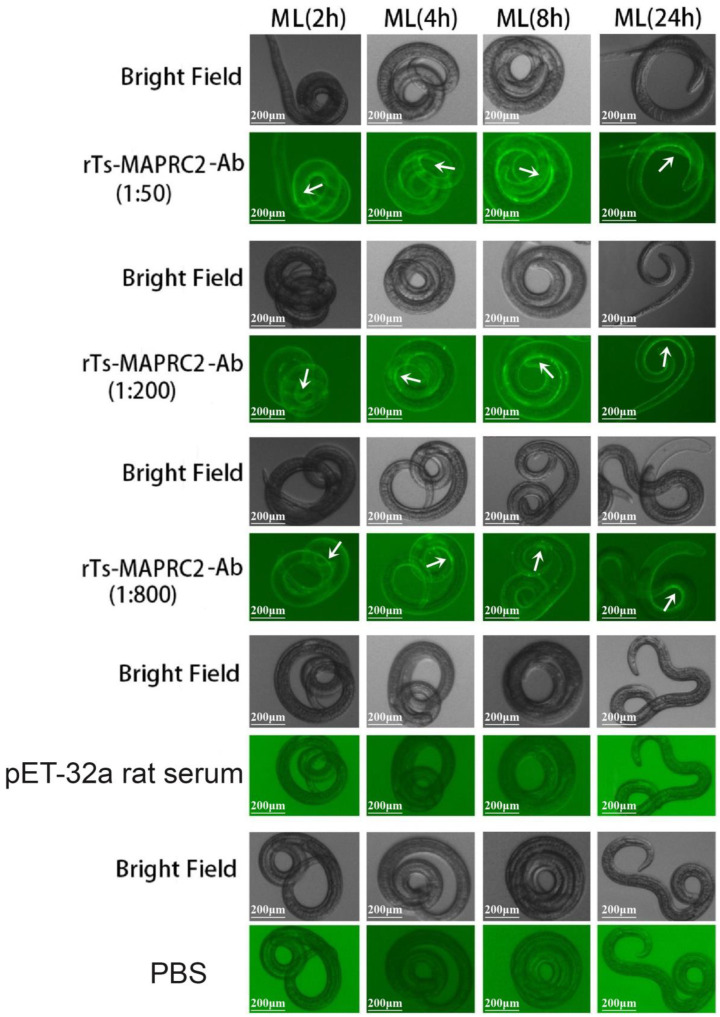
Identification of native *Ts*-MAPRC2 protein on the surface of *T. spiralis* muscle larvae (ML) analyzed by IIFT using rat sera against rTs-MAPRC2 at different concentrations (1:50, 1:200, and 1:800) with different time intervals (2 h, 4 h, 8 h, and 24 h) compared with pET32a rat serum (control group) and PBS (control group). Scale bar = 200 μm. White arrows point to positive results.

**Figure 2:**
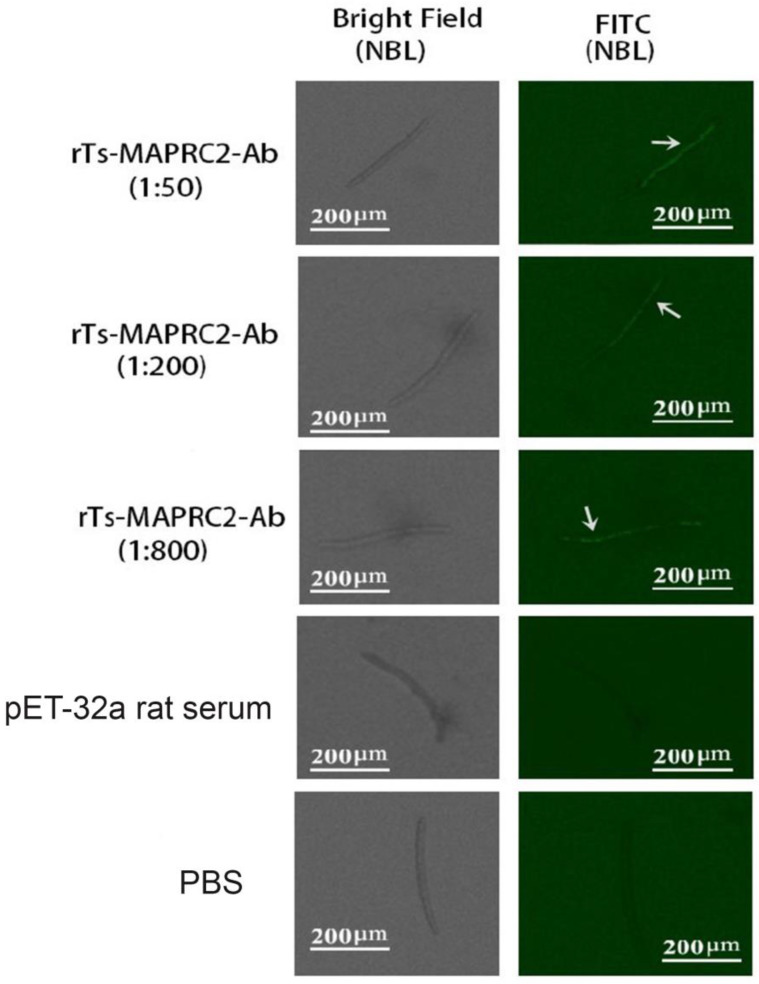
Identification of native Ts-MAPRC2 protein in *T. spiralis* newborn larvae (NBL) by IIFT using rat sera (at different concentrations) against rTs-MAPRC2. This was compared to pET32a rat serum (control group) and PBS (control group) after 24 h. Scale bar = 200 μm. White arrows point to positive results.

Anti-rTs-MAPRC2 rat serum was tested in different concentrations (1:50, 1:200, and 1:800) and different time intervals (2 h, 4 h, 8 h, and 24 h) on the cuticle at ML stage with pET32a rat serum (control group) and PBS (control group) (Figure 1). The results indicated that anti-rTs-MAPRC2 rat serum (1:50) was brighter than other concentrations (1:200 and 1:800). The FITC fluorescence protein is recognized more effectively at 8 h and 24 h (Figure S2). Furthermore, we also found native protein in newborn larvae (NBL) by using rat sera against rTs-MAPRC2 at different concentrations (1:50, 1:100, and 1:800) after 24 h in comparison to pET32a rat serum (control group) and PBS (control group) (Figure 2).

### 3.2. Effect of rTs-MAPRC2-Ab on ML and NBL In Vitro

**Figure 3:**
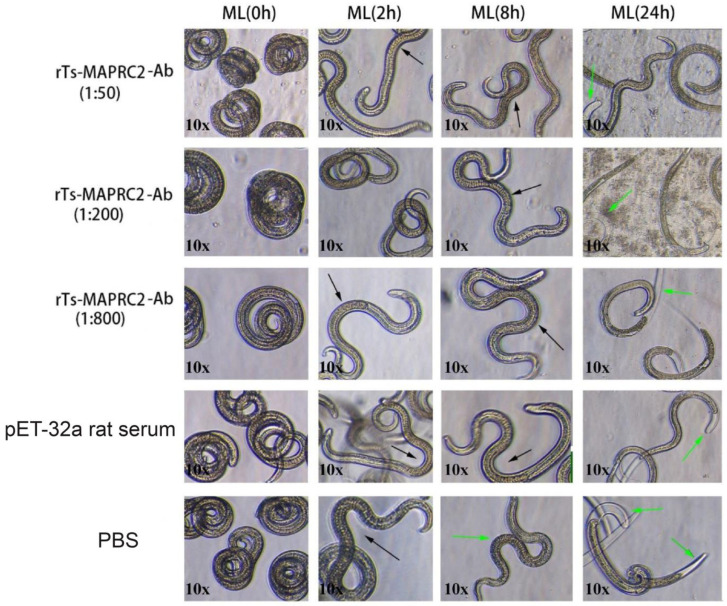
The selected concentration ratios of rTs-MAPRC2-Ab (1:50, 1:200, and 1:800) with varying time intervals, in conjunction with controls of pET32a rat serum (control group) and PBS (control group), were used at the ML stage to observe ecdysis (molting process) and motility at objective 10×. The green arrows point to ecdysis (molting process) and the black arrows represent motility.

At the ML stage, the selected concentration rTs-MAPRC2-Ab ratios (1:50, 1:200, and 1:800 dilutions) with various time intervals (0 h, 4 h, 8 h, 24 h) were used in conjunction with controls pET32a rat serum (control group) and PBS (control group). At all time intervals (0 h, 4 h, 8 h, 24 h) motility was present in the ML stage (Figure 3). The comparison of ecdysis (molting process) showed that after 24 h, the ecdysis process was slower in 1:50 (rTs-MAPRC2-Ab ratio) compared to 1:200 and 1:800 (rTs-MAPRC2-Ab ratio). In both pET32a rat serum (control group) and PBS (control group), the process of ecdysis was faster (Figure S3). In addition, we observed motility in newborn larvae (NBL) using rat sera against rTs-MAPRC2, at different concentrations (1:50, 1:100, and 1:800) following 24 h of treatment, when compared with pET32a rat serum (control group) and PBS (control group) (Figure S1). The results show that NBL worms were more motile in the control groups (pET32a-control and PBS-control). At 1:50 (rTs-MAPRC2-Ab ratio), motility was slower than at 1:200 and 1:800 (rTs-MAPRC2-Ab ratio).

### 3.3. Assessing the Infectivity of ML Treated with rTs-MAPRC2-Ab In Vivo

**Figure 4:**
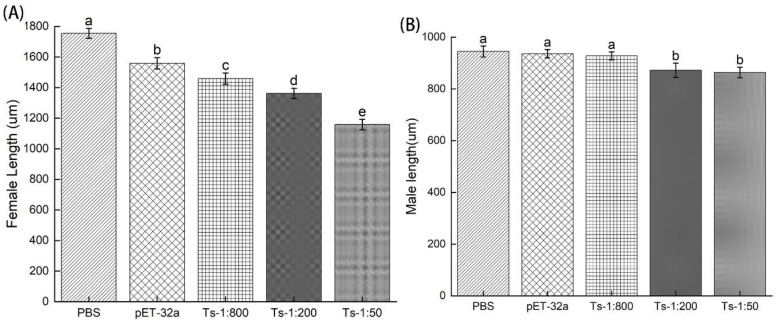
Length of *T. spiralis* from mice treated with rTs-MAPRC2-Ab (1:50, 1:200, and 1:800 dilutions), pET32a rat serum (control group) or PBS (control group) at six days post-infection (6 dpi). (**A**) Length of female adult worms (µm). (**B**) Length of male adult worms (µm). Statistical data are presented as mean ± SD. *p* ≤ 0.05 was considered significant. Different letters mean significant and the same letters mean nonsignificant.

The effects of rTs-MAPRC2-Ab on the infectivity and efficacy of ML were tested by infecting 500 MLs orally treated with rTs-MAPRC2-Ab (1:50, 1:200, and 1:800 dilutions), pET32a rat serum (control group) and PBS (control group). Five mice from each group were euthanized at 6 days post-infection and female adult worms were collected. Female adult worms treated with rTs-MAPRC2-Ab (1:50, 1:200, and 1:800 dilutions) were consistently shorter than worms from the pET32a rat serum and PBS groups (*p* ≤ 0.05). Furthermore, the adult female length treated with anti-rTs-MAPRC2 rat serum (1:50) was smaller than with the other concentrations (1:200, and 1:800) (*p* ≤ 0.05) (Figure 4A). A comparison of male adult worm groups (1:50, 1:200, and 1:800 dilutions) with pET32a rat serum and PBS showed some difference but no more significant a variation in length than that seen in female worms. (*p* ≤ 0.05) (Figure 4B). 

**Figure 5:**
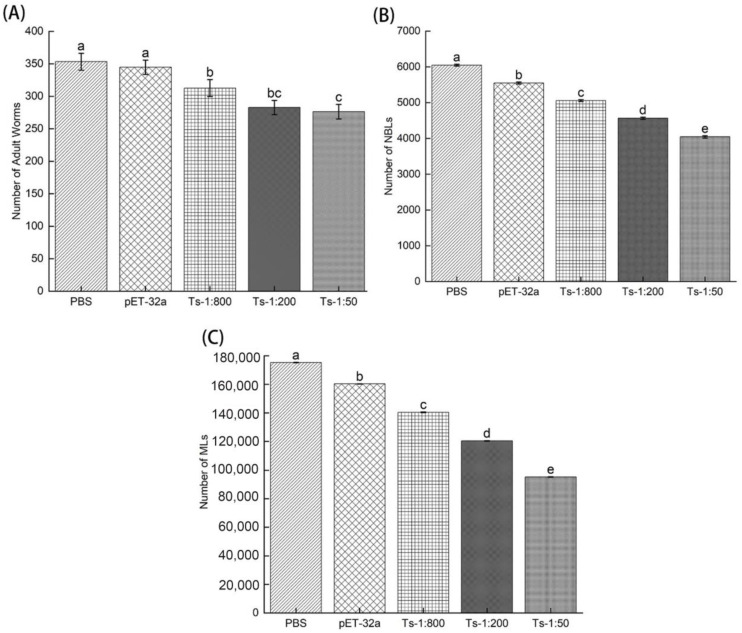
(**A**) number of adult worms collected after 6 dpi (n = 5), (**B**) the total number of newborn larvae collected after 24 h, and (**C**) the total number of muscle larvae collected after 35 days post-infection treated with rTs-MAPRC2-Ab (1:50, 1:200, and 1:800 dilutions), pET32a rat serum (control group), and PBS (control group) (n = 5). Statistical data are presented as mean ± SD. *p* ≤ 0.05 was considered significant. Different letters mean significant and the same letters mean nonsignificant.

Figure 5 shows the count of total adult worms after 6 dpi, collection of total newborn larvae (NBL) after 24 h, and the total number of muscle larvae (MLs) after 35 days post-infection (35 dpi) treated with rTs-MAPRC2-Ab (1:50, 1:200, and 1:800 dilutions), pET32a rat serum (control group) and PBS (control group). Figure 5A shows that adult worm burden was almost equal at 1:50 (rTs-MAPRC2-Ab ratio) and 1:200 but less than that at 1:800 (rTs-MAPRC2-Ab ratio), as well as in comparison to pET32a rat serum (control group) and PBS (control group) (*p* ≤ 0.05). Figure 5B shows the total number of newborn larvae collected from adult worms due to in vitro culture after 24 h. Results indicated that the total newborn larvae (NBLs) were lower in 1:50 (rTs-MAPRC2-Ab ratio) than in 1:200 and 1:800 (rTs-MAPRC2-Ab ratio), as well as in comparison to pET32a rat serum (control group) and PBS (control group) (*p* ≤ 0.05). Figure 5C shows the total number of muscle larvae collected (MLs) after 35 days post-infection (35 dpi). According to the results, the total number of muscle larvae collected (MLs) was lower in 1:50 (rTs-MAPRC2-Ab ratio) compared to 1:200 and 1:800 (rTs-MAPRC2-Ab ratio), as well as compared to pET32a rat serum (control group) and PBS (control group) (*p* ≤ 0.05).

### 3.4. Infectivity of NBL Treated with rTs-MAPRC2-Ab

**Figure 6:**
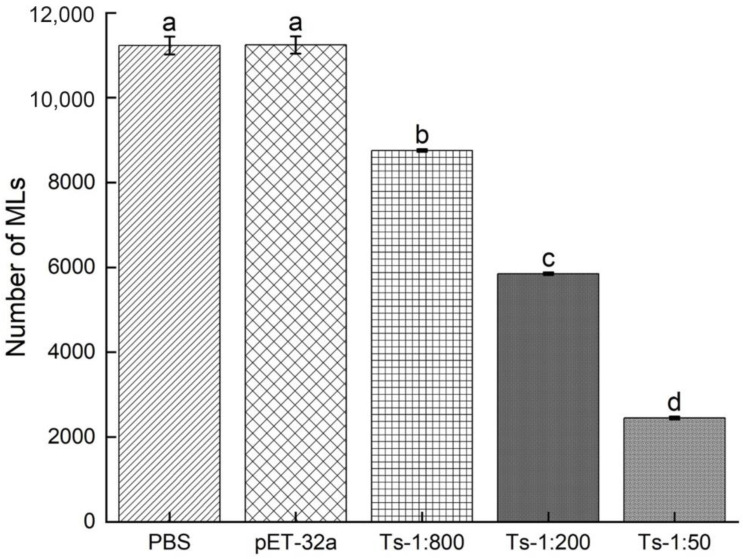
The total number of muscle larvae collected after 35 days post-infection (35 dpi) treated NBL with rTs-MAPRC2-Ab (1:50, 1:200, and 1:800 dilutions), pET32a rat serum (control group), and PBS (control group) (n = 5). Statistical data are presented as mean ± SD. *p* ≤ 0.05 was considered significant. Different letters mean significant and the same letters mean nonsignificant.

The NBLs were treated with rTs-MAPRC2-Ab (1:50, 1:200, and 1:800 dilutions), pET32a rat serum (control group), and PBS (control group). The total number of muscle larvae (MLs) was counted at 35 days post-infection (dpi). As shown in Figure 6, the total number of muscle larvae collected (MLs) was significantly lower in 1:50 (rTs-MAPRC2-Ab ratio) compared to in 1:200 and 1:800, and pET32a rat serum (control group) and PBS (control group) (*p* ≤ 0.05).

### 3.5. Specific siRNA-Mediated Suppression of Ts-MAPRC2 Protein Expression

**Figure 7:**
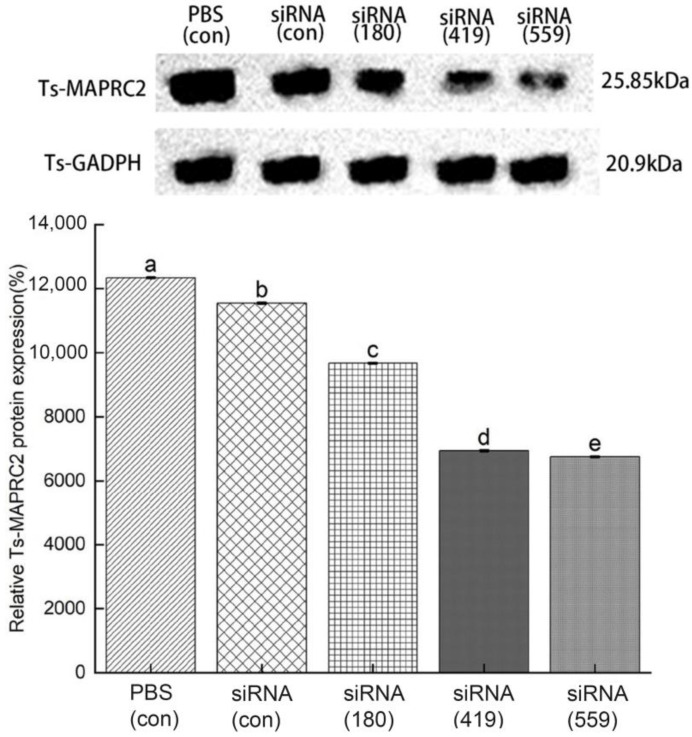
**Western blot analysis of Ts-MAPRC2 protein expression induced by siRNA.** Western blot with anti-rTs-MAPRC2 antibodies showed the specific inhibition of Ts-MAPRC2 protein expression of *T. spiralis* muscle larvae (ML) with different siRNA-treated groups (siRNA-180, siRNA-419, siRNA-559, siRNA-Control, and PBS control) for 3 days compared with internal control (Ts-GADPH). Protein expression densities were measured by Image J software and expressed in percentages. The tests were conducted in triplicate. Data are presented as the mean ± SD. *p* ≤ 0.05 was considered significant. Different letters indicate significance, while the same letters indicate nonsignificance.

ML were incubated in 2 μM siRNA-Ts-MAPRC2 of all treated groups (siRNA-180, siRNA-419, siRNA-559, siRNA-Control, and PBS control) for 3 days to determine the level of inhibition expression. siRNA-559 was found to have a greater inhibitory effect on Ts-MAPRC2 protein expression compared with siRNA-180 and siRNA-419. Comparing siRNA-559 and siRNA-419, siRNA-559 has more effect on silencing Ts-MAPRC2 protein expression (Figure 7) (*p* ≤ 0.05).

### 3.6. Specific siRNA-Mediated Suppression of Ts-MAPRC2 mRNA Expression

**Figure 8:**
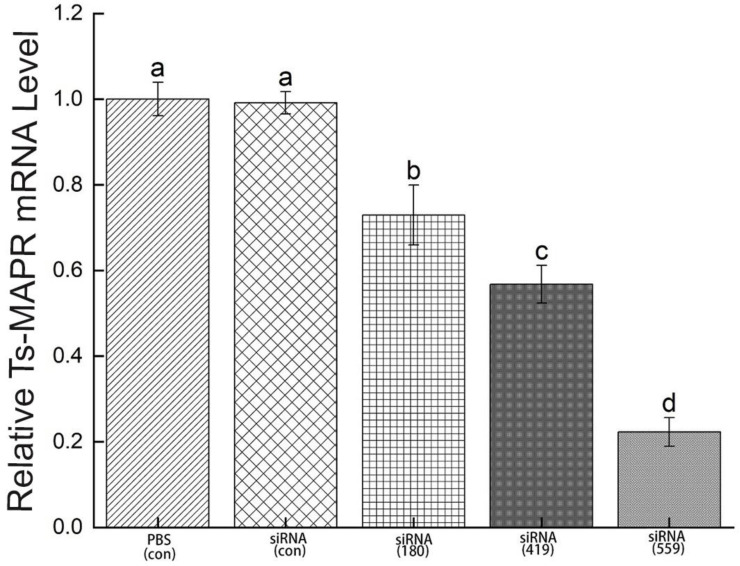
**mRNA level of Ts-MAPRC2 gene induced by siRNAs.** mRNA expression of the Ts-MAPRC2 gene of *T. spiralis* muscle larvae (ML) treated with different siRNAs (siRNA-180, siRNA-419, siRNA-559, siRNA-Control, and PBS control) for 3 days. Triplicates of the tests were performed. The data are presented as the mean ± SD. *p* ≤ 0.05 was considered significant. The same letter indicates insignificance, but a different letter indicates significance.

In order to determine mRNA expression, ML were incubated in 2 μM siRNA-Ts-MAPRC2 of the treated groups (siRNA-180, siRNA-419, siRNA-559, siRNA-Control, and PBS control) for 3 days. The results showed that siRNA-559 had a greater downregulation of the Ts-MAPRC2 gene than siRNA-419 and siRNA-180. In comparison to siRNA-419, siRNA-559 has significantly more downregulation of the Ts-MAPRC2 gene (*p* ≤ 0.05). Control siRNA and control PBS did not significantly differ in terms of mRNA expression (Figure 8) (*p* ≤ 0.05).

### 3.7. In Vitro Phenotyping of siRNA Effects on ML

**Figure 9:**
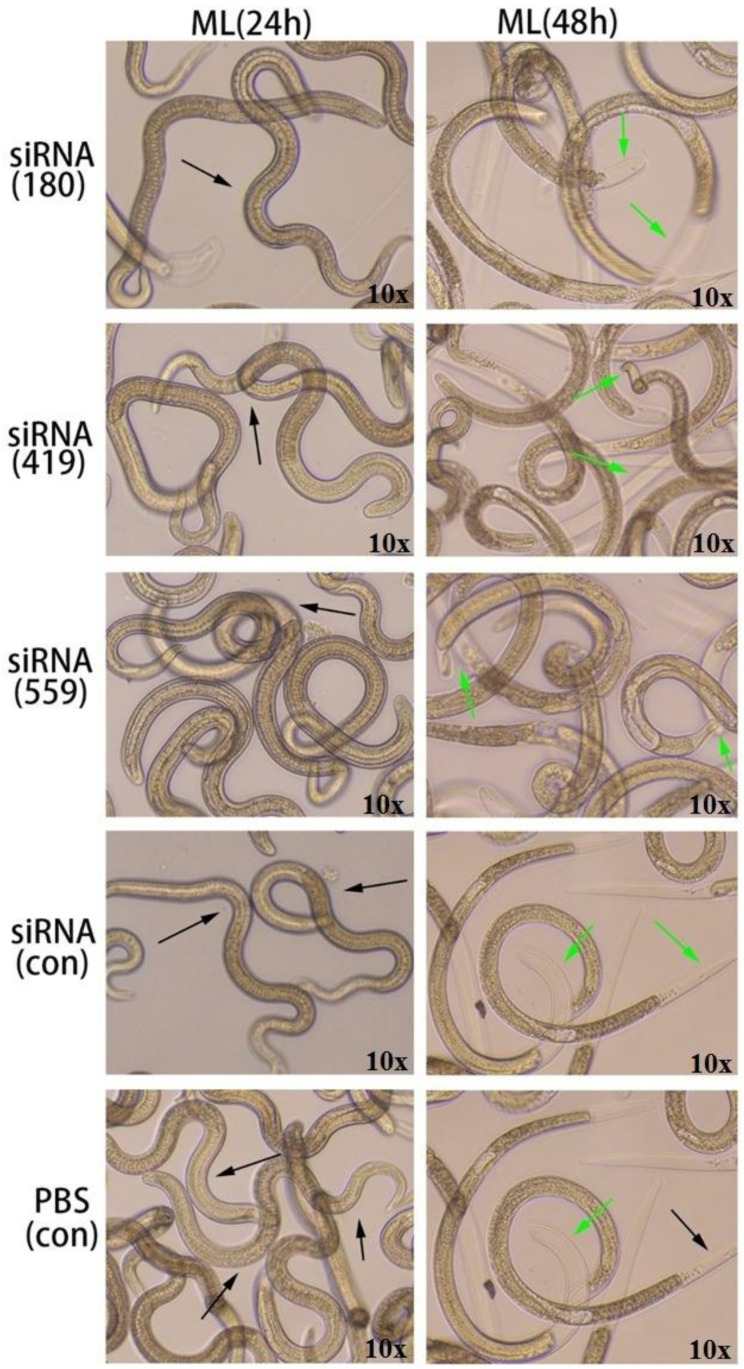
Various siRNAs targeting the Ts-MAPRC2 gene (siRNA-180, siRNA-419, siRNA-559) were used with varying time intervals (24 h, 48 h), in conjunction with siRNA-Control and PBS controls at the ML stage to observe motility and ecdysis (molting process) at objective 10×. The green arrows point to ecdysis (molting process) and the black arrows represent motility.

In the ML stage, the selected siRNAs (siRNA-180, siRNA-419, siRNA-559) were treated for 24 h and 48 h along with the controls (siRNA-control and PBS control). After 24 h, ecdysis (molting process) was slower in all treated groups (siRNA180, siRNA419, and siRNA559) as well as controls (siRNA-Control and PBS control). However, at 48 h, there was a difference in the ecdysis of all treated groups (siRNA180, siRNA419, siRNA559) as well as in the control groups (siRNA-Control and PBS control). Results showed that the siRNA-559 group had less ecdysis than the siRNA-419 and siRNA-180 groups. Compared to siRNA-419, siRNA-559 underwent a slower ecdysis process. The ecdysis of siRNA-180, control siRNA, and control PBS were normal (Figure 9).

### 3.8. Evaluation of siRNA-Treated NBL for Infectivity

**Figure 10:**
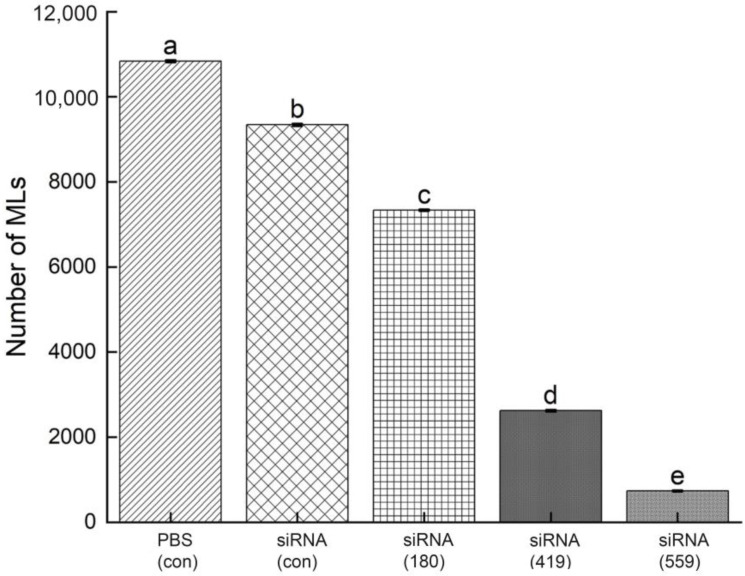
After 35 days post-infection (35 dpi), total muscle larvae were collected from NBLs treated with siRNAs (siRNA-180, siRNA-419, siRNA-559, siRNA-Control, and PBS). Statistical data are presented as mean ± SD. *p* ≤ 0.05 was considered significant. The same letters indicate significance, while different letters indicate nonsignificance.

NBLs treated with siRNAs (siRNA-180, siRNA-419, siRNA-559, siRNA-Control, and PBS control) were injected into mice through the tail vein and the total number of muscle larvae (MLs) were counted at 35 dpi. There was a significant decrease in muscle larvae (MLs) collected from the siRNA-559 group compared to the siRNA-419 and siRNA-180 groups. Compared to siRNA-419, siRNA-559 had a significantly lower total number of muscle larvae (MLs) (Figure 10) (*p* ≤ 0.05).

## 4. Discussion

PGRMC1 and PGRMC2 are members of the same family of membrane-associated progesterone receptors (MAPR) [43,44]. PGRMC-1 protein (28 kDa) was first isolated from pork smooth muscle [45,46]. In our previous study, we characterized MAPRC2 from *T. spiralis* and studied its interaction with progesterone and mifepristone [24,25]. In this study, we performed IIFT to find the native protein on the cuticle at MLat time intervals of 2 h, 4 h, 8 h, and 24 h (Figure 1). FITC fluorescence protein recognition increases at time intervals of 8 h and 24 h. Additionally, the native protein was also detected in newborn larvae (NBL) using rat antisera against the rTs-MAPRC2 at different concentrations after 24 h. (Figure 2). As a second group of studies, immunofluorescence assays were also performed to examine membrane-bound progesterone proteins in the *T. solium* cysticerci as well as in *T. spiralis* [22,24,47].

Moreover, motility and ecdysis (molting process) were observed with various concentrations of rTs-MAPRC2-Ab. The motility was present in the ML stage at all four time intervals (0 h, 4 h, 8 h, 24 h). The study of ecdysis (molting process) revealed that 1:50 (rTs-MAPRC2-Ab ratio) had a slower rate of molting compared to 1:200 (rTs-MAPRC2-Ab ratio) and 1:800 (rTs-MAPRC2-Ab ratio) (Figure 3). We also observed motility in newborn larvae (NBL) after treatments with different concentrations of anti-rat sera against rTs-MAPRC2 after 24 h of treatment (Figure S1). Motility was slower in 1:50 (rTs-MAPRC2-Ab ratio) than in 1:200 and 1:800 (rTs-MAPRC2-Ab ratio). Our results on motility and ecdysis (molting process) were correspondingly supported by the findings of Aleem et al. as well as Gagliardo et al. as reported in the literature [24,35,38,48].

Furthermore, testing the infectivity of ML in vivo after treatment with rTs-MAPRC2-Ab, adult female worms collected at 6 days post-immunization from groups immunized with rTs-MAPRC2-Ab (1:50, 1:200, and 1:800 dilutions) were consistently shorter than those from groups immunized with pET32a rat serum and PBS (*p* ≤ 0.05). It should be noted that the efficacy of 1:50 anti-rTs-MAPRC2 rat serum is superior to other concentrations (1:200 and 1:800) (Figure 4A) (*p* ≤ 0.05). In some respects, these findings are similar to those of Cui et al. [49].

Figure 5A shows adult worm burden was lower in 1:50 (rTs-MAPRC2-Ab ratio) than in 1:200 and 1:800 (rTs-MAPRC2-Ab ratio) (*p* ≤ 0.05). Figure 5B shows the total number of new larvae born from adult worms due to in vitro culture after 24 h. Overall, the number of newborn larvae (NBLs) was lower at 1:50 (rTs-MAPRC2-Ab ratio) than at 1:200 and 1:800 (rTs-MAPRC2-Ab ratio) (*p* ≤ 0.05). There was a lower number of muscle larvae collected (MLs) in 1:50 (rTS-MAPRC2-Ab) compared to 1:200 (rTS-MAPRC2-Ab) and 1:800 (rTS-MAPRC2-Ab) (Figure 5C) (*p* ≤ 0.05). Based on these results, it is evident that anti-rTs-MAPRC2 rat serum (1:50) provides a better result than other concentrations (1:200 and 1:800) in accordance with findings in the literature [33,49].

We subsequently analyzed the infectivity of NBLs treated with rTs-MAPRC2Ab. Based on the results (Figure 6), the number of muscle larvae collected (MLs) was significantly lower at 1:50 (rTs-MAPRC2-Ab ratio) (p ≤ 0.05). Several other studies also support these results, including Fei et al., Gagliardo et al., and Cui et al. [35,36,49].

siRNAs are artificially synthesized to study the host–parasite interaction and their application has proved an effective tool for identifying as well as studying gene functions [29,30]. Genes needed for parasite development and the growth process of *T. spiralis* can be silenced, which reduces or prevents parasite attacks on humans as well as on other mammals [31]. According to the results shown in Figure 7, siRNA-559 inhibits the expression of Ts-MAPRC2 more effectively than siRNA-180 and siRNA-419. Simultaneously, siRNA-559 significantly inhibits the Ts-MAPRC2 protein more effectively than siRNA-419 (*p* ≤ 0.05). This study supports the hypothesis that siRNA-Ts-MAPRC2 effects on worm reproduction might be mediated by specific steroid-binding domains in Ts-MAPRC2 [24,36,43]. In subsequent experiments, mRNA levels in ML were determined for all the treated groups. Compared to siRNA-419 and siRNA-180, siRNA-559 showed a greater downregulation of the Ts-MAPRC2 gene. Simultaneously with siRNA-419, siRNA-559 was found to downregulate the Ts-MAPRC2 gene more than siRNA-419 (*p* ≤ 0.05) (Figure 8). This indicates that suppression of Ts-MAPRC2 expression would have a detrimental effect on *T. spiralis* within the host [24,28,36]. Moreover, the selected siRNAs (siRNA-180, siRNA-419, siRNA-559) were exposed for 24 h and 48 h in order to observe motility and ecdysis (molting process). All three treated groups exhibited a slower ecdysis (molting process) after 24 h. At 48 h, all the treated groups showed a difference in the ecdysis. Based on the results, siRNA-559 showed a lower ecdysis rate than both siRNA-419 and siRNA-180. As shown in Figure 9, siRNA-180, control siRNA, and control PBS all gave normal results. Accordingly, the results of Aleem et al. and Gagliardo et al. also support our results on motility and ecdysis (molting process) [24,35]. Furthermore, we evaluated the infectivity of NBLs treated with siRNAs. According to the results shown in Figure 10, siRNA-559 significantly decreased the number of muscle larvae (MLs) collected compared to siRNA-419 and siRNA-180 (*p* ≤ 0.05). Other studies have supported these findings, including Fei et al., Gagliardo et al., and Cui et al. [24,35,36,44].

## 5. Conclusions

It is concluded from the present study that antibodies against Ts-MAPRC2 exerted a profound inhibitory effect on development in both muscle larvae (ML) and newborn larvae (NBL) of *T. spiralis*. All four time intervals (0 h, 4 h, 8 h, 24 h) in the ML stage and NBL (after 24 h) showed motility. For ecdysis (molting) in the ML stage, compared to 1:200 and 1:800 (rTs-MAPRC2-Ab ratios), 1:50 had a slower molting rate. Furthermore, siRNA-Ts-MAPRC2 strongly silenced the Ts-MAPRC2 gene in both muscle larvae (ML) and newborn larvae (NBL) of *T. spiralis*. With siRNA-559, MAPRC2 expression was downregulated with the strongest effect on parasite development. We found that larval survival, development, and infectivity were all significantly reduced. Based on these results, Ts-MAPRC2 could be considered a novel molecular target for the development of vaccines against *T. spiralis* infection.

## Figures and Tables

**Table 1:** The siRNAs used in the study.

siRNA Name	Sense (5′–3′)	Antisense (5′–3′)
**siRNA-180**	5′-CUGGGAUUCUUGCGGUAAUTT-3	5′-AUUACCGCAAGAAUCCCAGTT-3
**siRNA-419**	5′-GGUGGACCAUAUGGCUUAUTT-3′	5′-AUAAGCCAUAUGGUCCACCTT-3′
**siRNA-559**	5′-GGCUAUGCAUGAGCUGAAATT-3′	5′-UUUCAGCUCAUGCAUAGCCTT-3′
**siRNA-Control**	5′-UUCUCCGAACGUGUCACGUTT-3′	5′-ACGUGACACGUUCGGAGAATT-3′

## Data Availability

All data generated or analyzed during this study are included within the article.

## Supplementary Materials

The following supporting information can be downloaded at: https://www.mdpi.com/article/10.3390/vaccines11091437/s1, Figure S1: The concentration ratio of rTs-MAPRC2-Ab after 24 h, as well as controls of pET32a rat serum (control group) and PBS (control group), were used at the NBL stage to determine motility at objective 10×. The black arrow represented motility.; Figure S2: Relative flourescent intensity (%) into ML at different time intervals (**A**:2 h, **B**:4 h, **C**:8 h and **D**:24 h). Statistical data were presented as mean ± SD. *p* ≤ 0.05 was considered significant. Different letters mean significant and the same letters mean nonsignificant; Figure S3: Ecdysis process into ML at different time intervals (**A**:0 h, **B**:2 h, **C**:8 h and **D**:24 h). Statistical data were presented as mean ± SD. *p* ≤ 0.05 was considered significant. Different letters mean significant and the same letters mean nonsignificant; Figure S4: A. Western blot with anti-rTs-MAPRC2 antibodies showed the specific inhibition of Ts-MAPRC2 protein expression of *T. spiralis* muscle larvae (ML) with different siRNAs treated groups (siRNA-180, siRNA-419, siRNA-559, siRNA-Control, and PBS control). B. Compared with internal control (TsGADPH). C. Protein expression density were measured by Image J software and analysis were expressed in percentage. The tests were conducted in triplicate. Data was presented as the mean SD. *p* ≤ 0.05 was considered significant. Different letters indicate significance, while the same letters indicate nonsignificantly.
