# Supplementary material for: Inhibition of Trichinella spiralis Membrane-Associated Progesterone Receptor (MAPR) Results in a Reduction in Worm Burden
Source: Vaccines (Basel). 2023 Aug 31;11(9):1437. doi: 10.3390/vaccines11091437 (PMC10535220; doi:10.3390/vaccines11091437)
Supplement: Supplementary file 1 [file vaccines-11-01437-s001.zip › vaccines-2542890-supplementary.pdf]

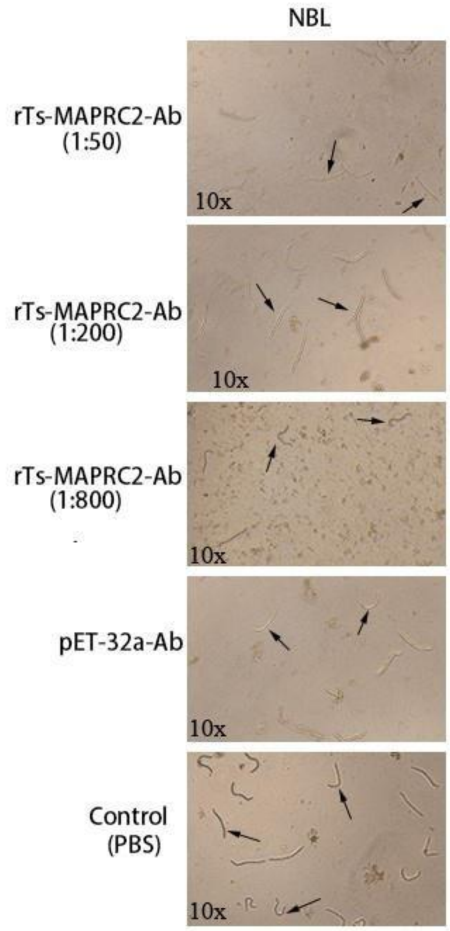

Figure S1. The concentration ratio of *rTs-MAPRC2-Ab* after 24h, as well as controls of pET32a rat serum (control group) and PBS (control group), were used at the NBL stage to determine motility at objective 10×. The black arrow represented motility.

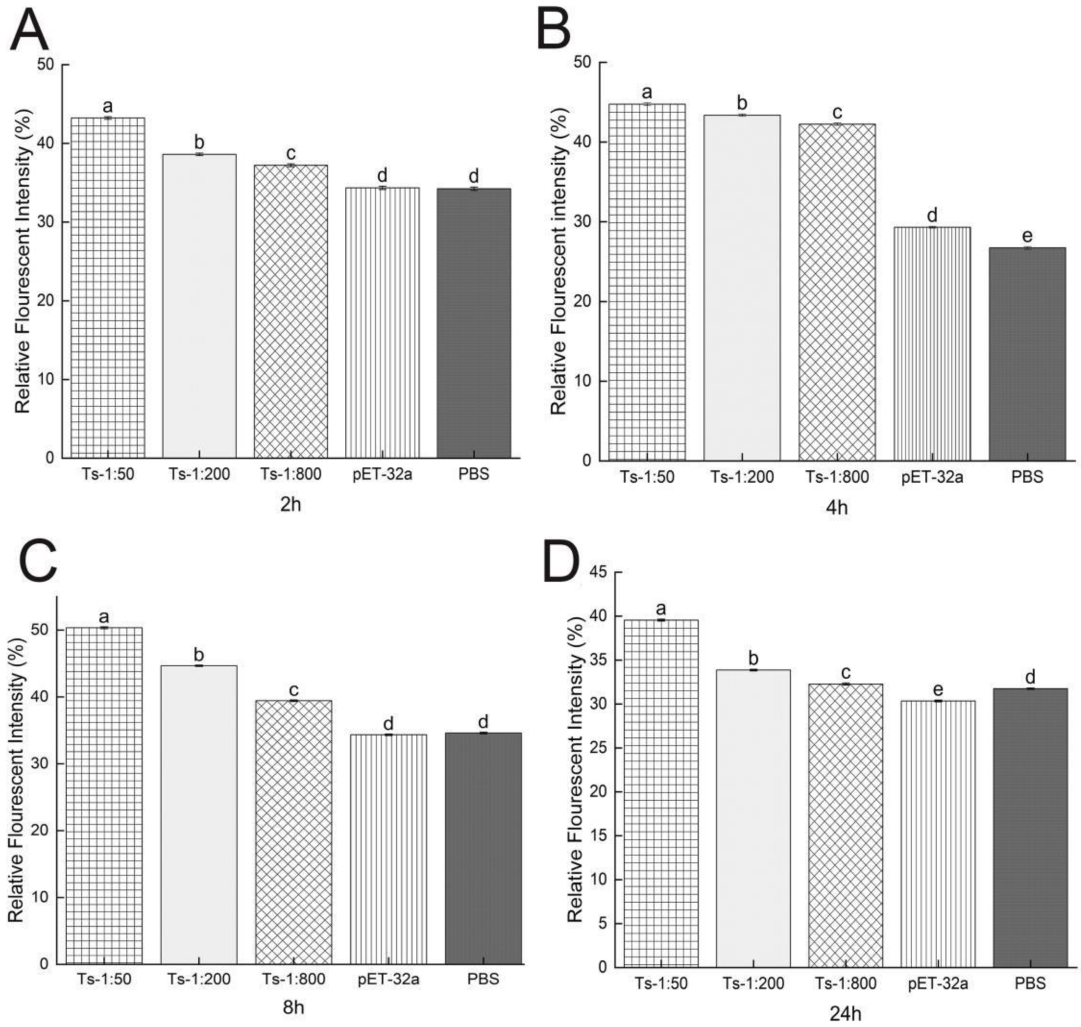

Figure S2. Relative flourescent intensity (%) into ML at different time intervals (**A**:2h, **B**:4h, **C**:8h and **D**:24h). Statistical data were presented as mean ± SD. $p \leq 0.05$ was considered significant. Different letters mean significant and the same letters mean nonsignificant.

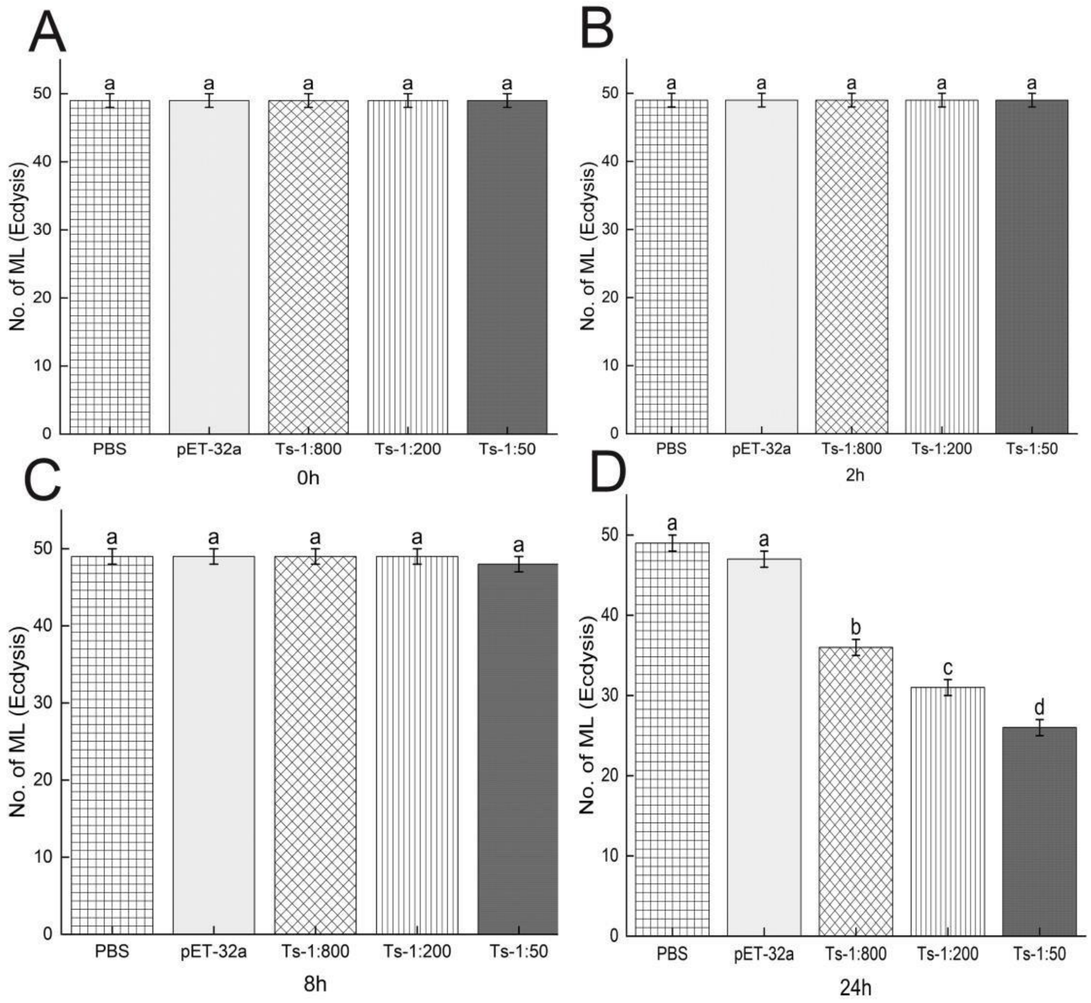

Figure S3. Ecdysis process into ML at different time intervals (**A**:0h, **B**:2h, **C**:8h and **D**:24h). Statistical data were presented as mean ± SD. $p \leq 0.05$ was considered significant. Different letters mean significant and the same letters mean nonsignificant.

Supplementary File (S)

Western Blot Picture with Protein Expression Density Analysis

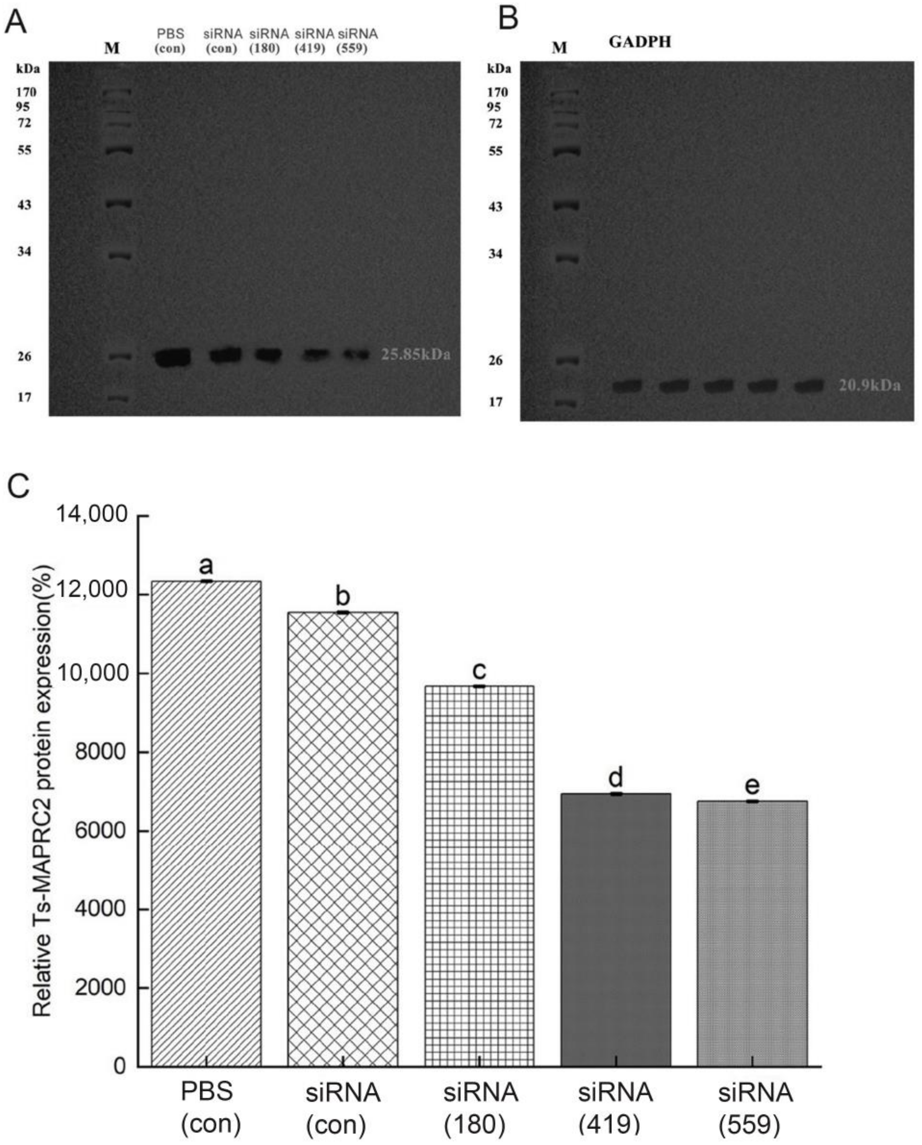

Fig S4. A. Western blot with anti-rTs-MAPRC2 antibodies showed the specific inhibition of Ts-MAPRC2 protein expression of *T. spiralis* muscle larvae (ML) with different siRNAs treated groups (siRNA-180, siRNA-419, siRNA-559, siRNA-Control, and PBS control). **Fig B.** Compared with internal control (TsGADPH). **Fig C.** Protein expression density were measured by Im-age J software and analysis were expressed in percentage. The tests were conducted in triplicate. Data was presented as the mean ± SD. $p \leq 0.05$ was considered significant. Different letters indi-cate significance, while the same letters indicate nonsignificantly.
